# The effect of gender, age, bodyweight, height and body mass index on plantar soft tissue stiffness

**DOI:** 10.1186/1757-1146-7-S1-A85

**Published:** 2014-04-08

**Authors:** Jee Chin Teoh, Taeyong Lee

**Affiliations:** 1 Department of Biomedical Engineering, National University of Singapore, Singapore

## Introduction

Foot abnormality has become a public health concern. Early detection of pathological soft tissue is hence an important preventive measure, especially to the elderly who generally have a higher risk of foot pathology (i.e. ulceration). However, the management of plantar tissue stiffness data is questionable.

The objective of this study is to assess the influence of gender and physical attributes such as height, weight and BMI on plantar soft tissue stiffness. It is also to evaluate whether it is necessary to isolate the differences in gender, age, bodyweight, height and body mass index in the data analysis procedure.

## Methods

100 healthy subjects were recruited from National Seoul University (SNU) hospital for the experiment. During stiffness measurement [1], indentor tip probes the plantar soft tissue to obtain localized force response underneath the 2nd metatarsal head pad at 3 different dorsiflexion angles of 0°, 20°, 40° and the hallux and heel at 0° Maximum tissue deformation is fixed at 5.6mm (close to literature data) [2].

Tissue behavior was characterized via K, stiffness constant.

T-tests were used to identify significant stiffness differences between left and right foot, as well as between male and female subjects on hallux and heel pad. Two-way ANOVA was used to analyze the data obtained from sub-MTH pad as the stiffness of the forefoot region. The level of significance was set at p<0.05. Pearson correlation was used to assess the relationship between bodyweight and BMI with plantar soft tissue stiffness.

## Results

The male and female participants were significantly varied in weight, height and BMI, but similar in age. There was a weak correlation for both the BW and BMI with plantar tissue stiffness (Table 1a and 1b). This showed that BW and BMI are unlikely the cause for the variation in stiffness data. Gender difference also did not show influence on stiffness measurement of plantar tissue at zero MTPJ flexion (Table 2).

**Table 1:** Pearson correlation for (a) body weight and (b) body mass index with plantar soft tissue stiffness

(a)			
Plantar location		Left	Right

Hallux		-0.1	-0.06
Heel		0.12	0.13
2nd MTH	0°	0.08	0.08
	20°	0.17	0.16
	30°	0.15	0.19

(b)			

Hallux		-0.09	0.02
Heel		-0.06	-0.16
2nd MTH	0°	-0.08	-0.08
	20°	-0.13	-0.14
	30°	-0.19	-0.12

**Table 2:** T-test results of plantar tissue stiffness due to gender difference

Plantar tissue stiffness		p
***Left***		
Hallux		0.72570177
Heel		0.21899688
2nd MTH	0°	0.48993505
	20°	0.05168678
	40°	0.01657689
		
***Right***		
Hallux		0.74204251
Heel		0.08408934
2nd MTH	0°	0.52305598
	20°	0.05769349
	40°	0.02280329

## Discussion

From the experimental results, it can be deduced that BW and BMI are weakly associated with plantar tissue stiffness and there was no significant difference in stiffness between male and female participants. No difference is found between left and right feet measurement. This suggests that normalizing of plantar tissue stiffness by either variable is not necessary. The data can be pooled and treated equally regardless of gender.
